# Hybrid Supervised and Reinforcement Learning for Motion-Sickness-Aware Path Tracking in Autonomous Vehicles

**DOI:** 10.3390/s25123695

**Published:** 2025-06-12

**Authors:** Yukang Lv, Yi Chen, Ziguo Chen, Yuze Fan, Yongchao Tao, Rui Zhao, Fei Gao

**Affiliations:** 1 College of Automotive Engineering, Jilin University, Changchun 130025, China; lvyk1522@mails.jlu.edu.cn (Y.L.); chenyi1522@mails.jlu.edu.cn (Y.C.); chenzg24@mails.jlu.edu.cn (Z.C.); fanyz23@mails.jlu.edu.cn (Y.F.); taoyc1522@mails.jlu.edu.cn (Y.T.); rzhao@jlu.edu.cn (R.Z.); 2 National Key Laboratory of Automotive Chassis Integration and Bionics, Jilin University, Changchun 130025, China

**Keywords:** autonomous vehicles, path tracking, motion sickness, supervised learning, reinforcement learning

## Abstract

Path tracking is an essential task for autonomous driving (AD), for which controllers are designed to issue commands so that vehicles will follow the path of upper-level decision planning properly to ensure operational safety, comfort, and efficiency. Current path-tracking methods still face challenges in balancing tracking accuracy with computational overhead, and more critically, lack consideration for Motion Sickness (MS) mitigation. However, as AD applications divert occupants’ attention to non-driving activities at varying degrees, MS in self-driving vehicles has been significantly exacerbated. This study presents a novel framework, the Hybrid Supervised–Reinforcement Learning (HSRL), designed to reduce passenger discomfort while achieving high-precision tracking performance with computational efficiency. The proposed HSRL employs expert data-guided supervised learning to rapidly optimize the path-tracking model, effectively mitigating the sample efficiency bottleneck inherent in pure Reinforcement Learning (RL). Simultaneously, the RL architecture integrates a passenger MS mechanism into a multi-objective reward function. This design enhances model robustness and control performance, achieving both high-precision tracking and passenger comfort optimization. Simulation experiments demonstrate that the HSRL significantly outperforms Proportional–Integral–Derivative (PID) and Model Predictive Control (MPC), achieving improved tracking accuracy and significantly reducing passengers’ cumulative Motion Sickness Dose Value (MSDV) across several test scenarios.

## 1. Introduction

With the rapid advancement of autonomous driving (AD) technology, autonomous vehicles are expected to deliver safer and more comfortable riding experiences [1,2]. AD systems typically consist of several core modules, including sensing [3], perception [4], decision [5], planning [6], and control [7]. Among them, the controller module serves as the core execution unit of the system and is responsible for translating decision-making plans into physical vehicle actions. Developing a real-time, accurate, stable, and comfortable tracking control system is critical for ensuring an exceptional passenger experience, yet achieving this goal remains challenging.

In path tracking, the lateral control of the tracking system should minimize tracking errors while restricting computational load to guarantee real-time performance and robustness. Furthermore, the system should be designed to alleviate potential Motion Sickness (MS) [8] experienced by passengers engaged in non-driving activities. MS is primarily attributed to sensory conflicts between visual and vestibular systems [9], and its severity can be quantified by the Motion Sickness Dose Value (MSDV) model [10]. Recent studies demonstrate that non-driving-related activities exacerbate MS by reducing passengers’ visual coupling with vehicle motion [11,12]. Consequently, the trade-off between precise, stable tracking control and reducing passengers’ MS remains an unresolved issue requiring urgent attention in autonomous vehicle development.

Researchers have developed various methods to address path-tracking challenges [13]. Proportional–Integral–Derivative (PID) controllers [14,15] remain popular due to their simple formulation and ease of maintenance. However, PID controllers are highly sensitive to direct feedback errors and require parameter retuning to adapt to varying scenarios, such as changing road conditions or complex path-tracking tasks. This necessitates repetitive gain scheduling, making PID controllers costly and time-consuming to calibrate and test.

Pure Pursuit (PP) controllers [16], based on geometric models, are widely adopted in real-world autonomous systems due to their structural simplicity. However, PP controllers lack predictive capability for future state variations, leading to trajectory oscillations in high-speed or high-curvature tracking scenarios. Their performance also depends heavily on appropriate look-ahead distance settings. To address this, more sophisticated algorithms (e.g., the Stanley method) [17] dynamically adjust look-ahead distances based on vehicle speed. However, such approaches fall under feedforward control and struggle to handle complex driving conditions requiring real-time vehicle feedback.

Other studies employ Linear Quadratic Regulator (LQR) methods [18] for path tracking, but their reliance on linearized vehicle models introduces significant simplifications, potentially compromising accuracy. Model predictive control (MPC) [19,20,21], which generates optimal control commands through iterative prediction horizons, offers improved performance. However, MPC’s precision is also affected by model simplifications, and its computational cost remains high due to the need to solve optimization problems iteratively at each time step, posing challenges for meeting real-time tracking requirements.

While recent advances show the integration of MS mitigation strategies in controller design for AD systems (e.g., [22]), most existing approaches still rely heavily on simplified linear models. This focus on model simplicity often overlooks the complex dynamics associated with MS effects, which can lead to suboptimal tracking performance and reduced passenger comfort. The lack of systematic frameworks to address MS mitigation remains a critical challenge to fully realize the potential of AD systems.

To address the inherent limitations of conventional controllers in adaptability and their failure to mitigate MS degradation, this study proposes a Hybrid Supervised– Reinforcement Learning (HSRL) framework. The framework starts with expert demonstration collection, followed by supervised pre-training of the initial policy through Behavior Cloning (BC). This phase ensures accelerated policy convergence and stabilized training dynamics by aligning the policy distribution with expert priors. Subsequently, an online Reinforcement Learning (RL) paradigm fine-tunes the policy via environmental interaction, enabling adaptation to stochastic scenarios while enhancing robustness and generalization.

The main contributions of this work can be summarized as follows:

(1) Dual-Stage Learning Mechanism: This study introduces a supervised pre-training-RL dual-stage optimization mechanism, integrating expert knowledge-guided supervised pre-training with the Twin Delayed Deep Deterministic Policy Gradient (TD3) algorithm. During the supervised pre-training phase, BC is employed to optimize the controllability of initial policy gradient update directions, effectively mitigating the inherent sample efficiency limitations of RL algorithms. This approach significantly reduces the resource consumption and training time required for model development. In the online RL phase, the pre-trained policy is further refined through iterative interaction, which enhances the model’s performance ceiling and generalization capability across diverse scenarios.

(2) MS Metric Integration: In this study, the reward mechanism of the RL algorithm innovatively integrates the standardized MSDV. Compared with traditional methods that rely solely on acceleration or vehicle jerk as evaluation metrics, this improvement significantly enhances the model’s optimization effectiveness in reducing passengers’ MS experience. By incorporating the MSDV as a multidimensional evaluation parameter into the reward function, the system can more precisely quantify MS-inducing factors in dynamic ride environments.

(3) Performance Evaluation: The effectiveness of the HSRL is validated through several simulation experiments, where the HSRL method is compared with PID and MPC controllers. The results demonstrate that the HSRL proposed in this study significantly outperforms other controller methods in tracking performance while reducing the MSDV by 15.7%.

The structure of this paper is as follows: Section 2 discusses and summarizes related work. Section 3 defines the problem and introduces our overall framework along with the HSRL method. Section 4 describes the experimental setup and compares the results of different methods. Section 5 concludes our work and presents future directions.

## 2. Related Works

### 2.1. Geometric Controllers

Geometric controllers, as classical methods in the field of autonomous vehicle path tracking, rely on vehicle kinematic models to establish analytical geometric constraints. Their core principle revolves around simplifying multi-wheel systems into equivalent single-track models through Ackermann steering configurations, generating steering control inputs via preview mechanisms or error feedback mechanisms [23]. Representative algorithms such as PP and the Stanley method achieve path tracking through arc fitting of preview points or lateral error compensation. Notably, Yunxiao Shan et al. [24] introduced an enhanced PP algorithm, substituting the conventional circular fitting approach with a clothoid curve to achieve greater path-fitting precision. Additionally, a fuzzy logic controller was implemented to dynamically optimize the look-ahead distance—a critical parameter dominantly affecting the PP algorithm’s control performance. Meanwhile, the gain parameters of the Stanley method can be optimized through neural dynamic programming for data-driven refinement [25].

Although geometric controllers are widely adopted in low-speed scenarios due to computational efficiency and parameter interpretability, their kinematic assumptions lead to significant dynamic coupling effects in high-speed conditions. And rigid geometric constraints tend to cause feedback lag and curvature discontinuities. More critically, this control strategy exclusively focuses on precise geometric path tracking without proactive adaptation to human perceptual dynamics, where operations like abrupt acceleration and frequent steering may exacerbate passengers’ MS symptoms [12].

### 2.2. Model-Free Controllers

Model-free controllers operate on the core principle of generating control inputs through direct mapping between feedback signals and preset control laws. As a classic model-free controller, PID dynamically adjusts steering angle outputs through error proportional, integral, and derivative terms. Their structural simplicity and real-time performance have enabled widespread adoption in autonomous vehicle steering control. For instance, Wael Farag [26] proposed a unique trial-and-error-based technique that employs the Cross-Track Error (CTE) as the sole input to the controller, with the output being the steering command. Park et al. [27] implemented high-precision steering actuator tracking using a PID architecture with dead-zone compensation, while Amer et al. [14] enhanced path tracking robustness through dual-loop PID design. Current research focuses on hybrid paradigms combining data-driven approaches with model-assisted techniques [28], such as introducing fuzzy logic to adaptively adjust PID gains or constructing dynamic optimization strategies for parameters based on RL. These innovations aim to enhance environmental adaptability while preserving the engineering convenience of model-free controllers, demonstrating potential advantages in low-speed scenarios with high uncertainty.

Despite achieving satisfactory performance through parameter fine-tuning, the PID method still faces inherent limitations due to the absence of feedforward compensation mechanisms for vehicle dynamic coupling and time-varying disturbances. Its parameter tuning heavily relies on empirical trial-and-error methods, while fixed gains struggle to adapt to nonlinear characteristics under complex operating conditions, such as sudden road adhesion changes and load transfers. Moreover, model-free controllers fundamentally lack active modeling capabilities for correlating human vestibular perception with vehicle motion dynamics [29]. They cannot suppress occupant sensory conflicts through dynamic adjustments of acceleration profiles or steering smoothness parameters, resulting in elevated MS risks induced by abrupt acceleration/deceleration or high-frequency lateral movements.

### 2.3. Model-Based Controllers

Model-based control methods construct control laws through the deep coupling of vehicle dynamic characteristics. The core principle is to utilize system state equations and optimal control theory for feedforward–feedback collaborative optimization [30]. Typical paradigms include MPC [31] and LQR. MPC generates control sequences that satisfy multiple constraint conditions by optimizing over a rolling time horizon. Jacob et al. reduced the computation time to 2 ms by using custom C code [32], significantly enhancing engineering feasibility. Merabti et al. [33] deployed Beal’s optimization method to solve the nonlinear problem of a vehicle. LQR, on the other hand, solves for the optimal gain analytically based on a quadratic cost function but requires path pre-sampling to compensate for its lack of foresight. The feedforward-feedback composite architecture proposed by Shladover et al. [34] innovatively combines path curvature feedforward with differential feedback of deviations, while the lateral offset tangent condition introduced by Kapania et al. [35] further enhances tracking robustness in extreme conditions. Current research focuses on simplifying model order reduction, data-driven model calibration, and hybrid control strategy design to balance theoretical rigor with engineering applicability.

Although these methods perform noticeably in terms of accuracy, their performance is constrained by model mismatches and computational complexity. And such methods cannot actively suppress sensory conflicts caused by sudden longitudinal acceleration or low-frequency lateral oscillations.

## 3. Methodology

### 3.1. Problem Definition

This study aims to resolve control challenges in autonomous driving by enabling real-time and seamless path tracking within traffic environments. The proposed control framework necessitates simultaneous minimization of trajectory deviations and mitigation of passengers’ MS perception. At each control update interval, the path-tracking system generates optimal control commands based on real-time vehicle motion states, waypoint coordinates, and road curvature parameters, thereby achieving a desired tracking performance that balances kinematic precision with passenger comfort requirements.

#### 3.1.1. Input Representation

**Figure 1:**
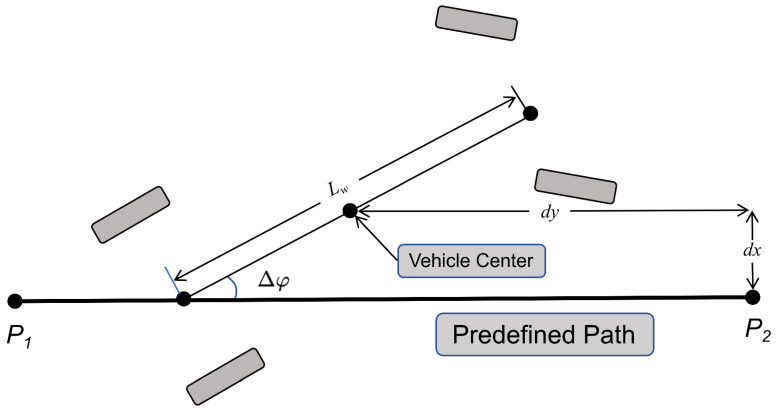
Definition of heading error $\left(\Delta \varphi \right),$ directional deviations (dx,dy), and wheelbase $\left(L_{w}\right)$.

A state space representation is proposed for HSRL as $S=\left\{dx,\;dy,\;\Delta \varphi ,\;v,\;k\right\}$, where each state variable is defined as follows:

- dx∈R and dy∈R denote the directional deviations (m) between the vehicle’s current position and reference path waypoint, as shown in Figure 1.
- $\Delta \varphi \in \left(-\pi ,\pi \right]\left(rad\right)$ quantifies the heading error, defined as the angular difference between the vehicle’s orientation and the target trajectory direction, as shown in Figure 1.
- *v* represents the vehicle’s longitudinal velocity.
- *k* denotes the curvature parameter $\left(m^{-1}\right)$ of the predefined reference path. By incorporating this parameter and explicitly quantifying the geometric characteristics of the path, the path-tracking model significantly enhances its adaptability to unstructured road geometries.

#### 3.1.2. Output Representation

The action space design $A=\left\{a_{acc},\delta_{steer}\right\}$ proposed in this study achieves both action space simplification and implicit encoding of physical constraints via piecewise linear mapping of longitudinal acceleration command and direct control of steering wheel angles. Specifically, the longitudinal acceleration command $a_{acc}\in \left[-a_{max},a_{max}\right]$ is bounded by a threshold $a_{th}=-0.5\cdot a_{max}$. Through extensive trials and validations, setting the coefficient of $a_{th}$ to 0.5 yields the most stable system response; deviating from this value (either smaller or larger) tends to introduce oscillatory behavior or control jitter. This is implemented via mutually exclusive brake/throttle piecewise linear functions, defined as Equation (1), which ensure smooth control transitions by avoiding abrupt switching between control modes. Meanwhile, $\delta_{steer}\in \left[-\delta_{max},\delta_{max}\right]$ directly governs the steering angle.

(1) $$\mathrm{brake}=\left\{\begin{array}{cc} 2(a_{max}+a_{acc}) & \mathrm{if}\;a_{acc}\leq a_{th}\\ 0 & \mathrm{otherwise} \end{array}\right.,\;\;\mathrm{throttle}=\left\{\begin{array}{cc} 0 & \mathrm{if}\;a_{acc}\leq a_{th}\\ \frac{2}{3}\left(a_{acc}+a_{th}\right) & \mathrm{otherwise} \end{array}\right.$$

### 3.2. Method Framework

**Figure 2:**
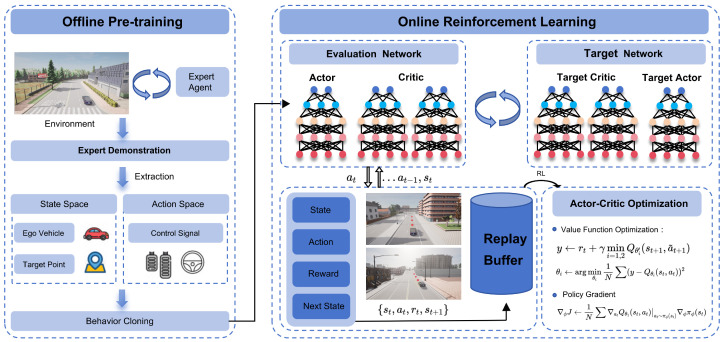
The overall framework of the proposed two-stage training. It combines offline supervised learning and RL to enhance training stability.

The HSRL framework is proposed in this section with a two-stage mechanism to balance path tracking accuracy with passenger MS mitigation, comprising (1) an offline supervised learning phase for initial policy and (2) an online RL phase for adaptive optimization. The architectural schematic of the proposed framework is illustrated in Figure 2.

During the offline supervised learning phase, an expert agent is employed to collect a trajectory dataset within the target environment, capturing expert demonstrations for policy initialization. Subsequently, the initial policy network is trained via supervised learning using BC, enabling the acquisition of static mapping relationships inherent in the expert strategies [36].

Following offline supervised learning, an Actor–Critic architecture is further refined using online interaction data from RL. By incorporating exploration noise and temporal difference learning to optimize long-term returns while preserving expert prior knowledge, this approach achieves a seamless transition from supervised learning to autonomous path tracking. The hybrid paradigm significantly enhances training efficiency and policy stability while substantially reducing temporal training overhead.

### 3.3. Offline Supervised Learning

**Figure 3:**
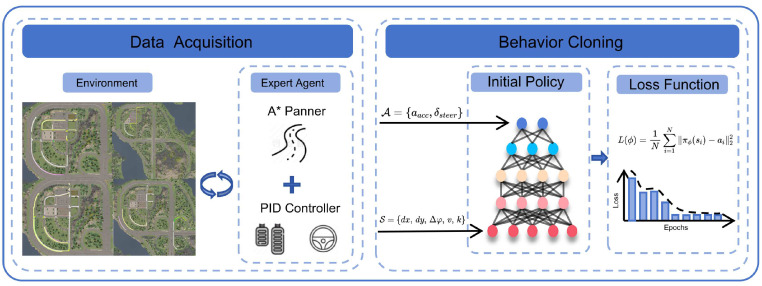
Supervised learning for initial policy.

During the supervised learning phase, the expert agent collects a dataset containing state–action pairs, where the state space S  comprises multi-dimensional continuous features, and the action space A represents continuous control signals. The supervised learning process is illustrated in Figure 3.

#### 3.3.1. Expert Demonstration

The expert agent performs tasks within the widely adopted Longest6 benchmark [37] in autonomous driving to collect datasets. In this study, Town04 was selected for agent data collection due to its road environment complexity. The expert agent is composed of an A* planner and two PID controllers (lateral and longitudinal) to generate high-quality datasets.

The A* planner [37] receives a discrete coordinate sequence from the Longest6 benchmark as input and generates a high-density trajectory sequence as output. By leveraging the topological structure of the CARLA map, it creates continuous road-compliant paths between sparse waypoints through a systematic search process. After obtaining the high-density trajectory sequence generated by the A* planner, the lateral PID controller calculates the lateral control error $e_{t}^{\mathrm{lat}}$ based on the extracted position coordinates from the trajectory. The longitudinal PID controller computes the longitudinal control error $e_{t}^{\mathrm{lon}}$ using the target speed (configured via speed settings) and the current vehicle speed. In this study, the speed configuration is adjustable within a range of 10–130 km/h. Specifically, the $e_{t}^{\mathrm{lon}}$ and $e_{t}^{\mathrm{lat}}$ are calculated as follows:

(2) $$\begin{array}{cc} e_{t}^{\mathrm{lat}} & =\arccos\left(\frac{{\vec{\omega }}_{t}\cdot {\vec{v}}_{t}}{∥{\vec{\omega }}_{t}∥∥{\vec{v}}_{t}∥}\right) \end{array}$$

(3) $$\begin{array}{cc} e_{t}^{\mathrm{lon}} & =v_{t}^{\mathrm{target}}-v_{t} \end{array}$$

Here, ${\vec{\omega }}_{t}$ represents the vector from the current position of vehicle to its next closest waypoint, which is derived from the high-density trajectory sequence generated by the A* planner. ${\vec{v}}_{t}$, $v_{t}^{\mathrm{target}}$, and $v_{t}$ represent the velocity vector, the desired velocity, and current velocity of vehicle, respectively. Then, the lateral and longitudinal control output $\delta_{steer}$ and $a_{acc}$ are computed separately using PID controllers. Then, the control outputs were saved as our action space $A=\left\{a_{acc},\delta_{steer}\right\}$.

(4) $$\begin{array}{cc} \delta_{steer} & =K_{P}^{\mathrm{lat}}\cdot e_{t}^{\mathrm{lat}}+K_{I}^{\mathrm{lat}}\cdot \int_{0}^{t}e_{x}^{\mathrm{lat}}\;dx+K_{D}^{\mathrm{lat}}\cdot \frac{d}{dt}e_{t}^{\mathrm{lat}} \end{array}$$

(5) $$\begin{array}{cc} a_{acc} & =K_{P}^{\mathrm{lon}}\cdot e_{t}^{\mathrm{lon}}+K_{I}^{\mathrm{lon}}\cdot \int_{0}^{t}e_{x}^{\mathrm{lon}}\;dx+K_{D}^{\mathrm{lon}}\cdot \frac{d}{dt}e_{t}^{\mathrm{lon}} \end{array}$$

where $K_{P}^{\mathrm{lon}}$, $K_{I}^{\mathrm{lon}}$, $K_{D}^{\mathrm{lon}}$ and $K_{P}^{\mathrm{lat}}$, $K_{I}^{\mathrm{lat}}$, $K_{D}^{\mathrm{lat}}$ represent the coefficients of the proportional, integral, and derivative terms in the lateral and longitudinal control, respectively.

To generate training data, the expert agent performs autonomous driving tasks in sequence along four routes provided by the Longest6 benchmark in Town04. During these tasks, required state space data $S=\left\{dx,\;dy,\;\Delta \varphi ,\;v,\;k\right\}$ is collected along the routes and combined with the action space $A=\left\{a_{acc},\delta_{steer}\right\}$ to form a state–action pair dataset.

The dataset containing state–action pairs is then randomly split into training and validation sets with a 4:1 ratio. The training set is used for policy network optimization via gradient descent, while the validation set monitors model generalization performance to prevent overfitting. To ensure consistent evaluation, the dataset partitioning employs stratified random sampling, which preserves the distribution characteristics of both states and actions.

#### 3.3.2. Loss Function

The optimization objective of BC is to minimize the discrepancy between policy network outputs and expert actions. This study employs Mean Squared Error (MSE) as the loss function, defined as

(6) $$L\left(\phi \right)=\frac{1}{N}\sum_{i=1}^{N}{∥\pi_{\phi }\left(s_{i}\right)-a_{i}∥}_{2}^{2}$$

where *N* denotes the batch size, $\pi_{\phi }\left(s_{i}\right)$ represents the action predicted by the initial policy network, and $a_{i}$ corresponds to the expert action. The policy parameters are iteratively updated via gradient descent as follows:

(7) $$\phi =\phi -\alpha \nabla_{\phi }L\left(\phi \right)$$

where α denotes learning rate, and $\nabla_{\phi }$ calculates the gradient with respect to policy parameters ϕ at the iteration *t*. The training process utilizes mini-batch gradient descent with a batch size of 64 and the Adam optimizer, initialized with a learning rate of $10^{-3}$. After each training epoch, the validation loss is computed by averaging the MSE over all validation samples, providing a robust indicator of model convergence. This optimization process is achieved through backpropagation, ensuring the progressive convergence of the initial policy’s action predictions toward the expert demonstration distribution. In continuous action spaces, the MSE directly quantifies the Euclidean distance between predicted actions and expert actions, with its convexity and smoothness facilitating gradient-based optimization.

### 3.4. Online RL Framework

**Figure 4:**
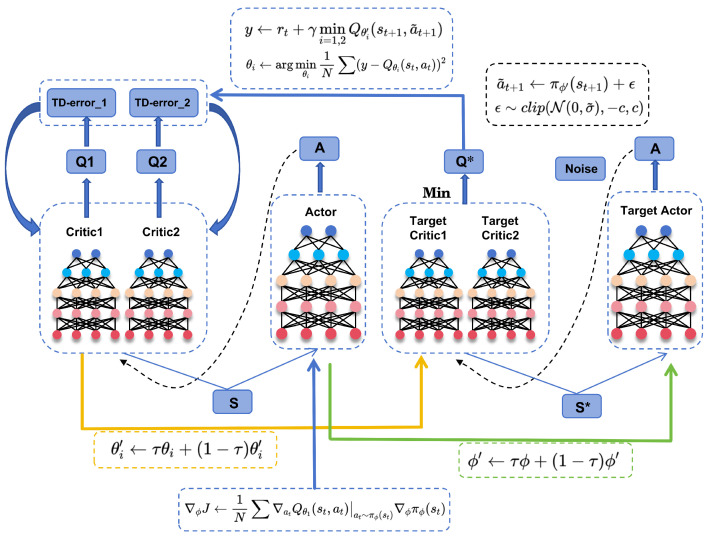
Architecture of TD3. The Q* represents the minimum output between the two target critic networks.

After completing offline supervised learning, the pre-trained policy is loaded, with two value networks initialized. Subsequently, the efficient TD3 algorithm [38] is adopted for policy updates. Specifically, it employs two independent critic networks and delayed updates for the actor network to mitigate issues such as Q-value overestimation and inherent, unstable policy updates. The architecture of the TD3-based algorithm in the proposed RL phase is illustrated in Figure 4.

#### 3.4.1. Markov Decision Process

In this phase, a sequential modeling approach is adopted, where the agent continuously interacts with the environment to acquire historical state–action information and perform actions at the current time step. This process is formalized as a standard MDP [39], defined as a tuple M=(S,A,P,r,γ), where S is the state space, $S\subseteq R^{n}$, containing instantaneous information about the interaction between the agent and the environment. A is the action space, $A\subseteq R^{m}$, representing the set of actions the agent can perform. Each action *a*∈A corresponds to a control command. P is the state transition probability function, $P:S\times A\times S\to \left[0,1\right]$. *r* is the reward function, and r:S×A→R. $\gamma \in \left[0,1\right]$ is the discount factor.

In the MDP framework, the goal of RL is to find an optimal policy $\pi_{\phi }$ that maximizes the cumulative reward. Specifically, the objective is to maximize the expected cumulative reward $J_{R}\left(\pi_{\phi }\right)$, which is the total expected reward over time. The state space and action space definitions in this section follow the definitions established in the problem statement and are consistent with those used in the offline supervised learning phase.

#### 3.4.2. Reward Function

To strike a balance between tracking performance and comfort, this section constructs a reward function architecture, encompassing components such as velocity, trajectory, heading, control, and MSDV rewards to support the optimization of the policy and accelerate convergence. The total reward is formulated as follows:

(8) $$R_{total}=R_{velocity}+R_{trajectory}+R_{heading}+R_{control}+R_{MSDV}$$

The velocity component $R_{velocity}$ evaluates speed management by rewarding proximity to the target velocity:

(9) $$R_{velocity}=\alpha_{1}\cdot \left(1-\frac{v_{ego}-v_{des}}{v_{max}}\right)$$

where $v_{ego},v_{des}$, and $v_{max}$ represent the current velocity, desired velocity, and maximum permissible speed, respectively. This encourages appropriate speed adaptation throughout the driving task. The trajectory component $R_{trajectory}$ evaluates the arrival of path points by measuring the displacement deviation in the *x* and *y* directions:

(10) $$R_{trajectory}=-\alpha_{2}\cdot \left(dx^{2}+dy^{2}\right)$$

The heading component $R_{heading}$ evaluates directional alignment with the intended route:

(11) $$R_{heading}=-\alpha_{3}\cdot \delta_{angular}$$

where $\delta_{angular}$ measures the absolute angular deviation between vehicle orientation and route direction. This ensures proper vehicle alignment along the planned path. The control component $R_{control}$ incentivizes smooth steering inputs:

(12) $$R_{control}=\left\{\begin{array}{cc} -\beta_{1} & \mathrm{if}\left|\delta_{steer}^{t}-\delta_{steer}^{t-1}\right|>\delta_{steer}^{threshold}\\ 0 & \mathrm{otherwise} \end{array}\right.$$

where $\delta_{steer}^{t}$ and $\delta_{steer}^{t-1}$ represent current and previous steering angles, with $\delta_{steer}^{threshold}$ set at 0.01. This discourages abrupt steering adjustments that could compromise ride comfort.

The MSDV is a metric that measures the accumulation of MS over time, as outlined in the ISO 2631 standard [10]. This metric accounts for sickening stimuli by applying frequency-dependent weightings to acceleration across different frequency ranges; this is because MS is influenced by the frequency of motion to which individuals are exposed [40]. The MSDV is defined as Equation (14):

(13) $$R_{MSDV}=-\beta_{2}\cdot MSDV$$

(14) $$MSDV=\sqrt{\int_{0}^{T}{\left[a_{x,w_{1}}\left(t\right)\right]}^{2}dt}\;\;+\sqrt{\int_{0}^{T}{\left[a_{y,w_{2}}\left(t\right)\right]}^{2}dt}$$

where $a_{x,w_{1}}\left(t\right)$ and $a_{y,w_{2}}\left(t\right)$ are weighted accelerations in longitudinal and lateral directions in time domain; dt is the time increment, and *T* is the exposure time. The MS frequency weighting curve proposed in [41] was adopted as the baseline. Through multiple iterations of revision and experimental validation, the optimal average weights for $a_{x,w_{1}}\left(t\right)$ and $a_{y,w_{2}}\left(t\right)$ were ultimately determined as 0.6 and 0.4, respectively. This parameter selection achieved dual objectives of minimizing weighting errors while maintaining computational simplicity in reward estimation.

#### 3.4.3. RL Optimization Process

In the online RL framework, the HSRL achieves policy optimization through continuous interaction with the dynamic environment. Its core objective is to maximize long-term cumulative returns. A dual critic network (Double Q-Learning) is introduced, which updates its parameters using temporal difference (TD) method, along with the pre-trained policy network $\pi_{\phi }$. The two target critic networks calculate the value of the next state:

(15) $$\left\{\begin{array}{cc} y_{1}=r+\gamma Q_{\theta_{1}^{\prime }}\left(s_{t+1},{\tilde{a}}_{t+1}\right) & \\ y_{2}=r+\gamma Q_{\theta_{2}^{\prime }}\left(s_{t+1},{\tilde{a}}_{t+1}\right) \end{array}\right.$$

where the ${\tilde{a}}_{t+1}$ represents the action generated by the target policy network and perturbed with clipped noise. In addition, to address the trade-off between bias and variance, the calculation of the q-value should be smoothed to avoid overfitting. Therefore, truncated normal distribution noise is added to each action as regularization, making the modified target update as follows:

(16) $${\tilde{a}}_{t+1}\leftarrow \pi_{\phi^{\prime }}\left(s_{t+1}\right)+\epsilon ,\epsilon ∼clip\left(N\left(0,\tilde{\sigma }\right),-c,c\right)$$

The minimum output value of the target network is selected as the target q-value to offset the overestimation problem of q-values. This is substituted into the Bellman Equation (19) to compute the TD-error and the loss function (18):

(17) $$y=r_{t}+\gamma \min_{i=1,2}Q_{\theta_{i}^{\prime }}\left(s_{t+1},{\tilde{a}}_{t+1}\right)$$

(18) $$L=\frac{1}{N}\sum {\left(y-Q_{\theta_{i}}\left(s_{t},a_{t}\right)\right)}^{2}$$

(19) $$Q_{\theta_{i}^{\prime }}\left(s_{t},a_{t}\right)=E\left[r\left(s_{t},a_{t}\right)+\gamma Q_{\theta_{i}^{\prime }}\left(s_{t},{\tilde{a}}_{t}\right)\right]$$

However, observations with errors are prone to causing divergence. Therefore, to minimize error propagation, the policy network is designed to update at a lower frequency than the critic network. That is, after multiple updates of the critic network, the policy network adjusts its parameters through gradient backpropagation. The lower the update frequency of the policy network, the smaller the variance in the q-value function updates, leading to a higher-quality policy. The parameter updates of the policy network are achieved through deterministic policy gradients. The specific loss gradient formula is as follows:

(20) $$\nabla_{\phi }J\leftarrow \frac{1}{N}\sum \nabla_{a_{t}}Q_{\theta_{1}}\left(s_{t},a_{t}\right){|}_{a_{t}∼\pi_{\phi }\left(s_{t}\right)}\nabla_{\phi }\pi_{\phi }\left(s_{t}\right)$$

### 3.5. Path-Tracking Algorithm Based on HSRL

In this section, the path-tracking algorithm based on HSRL is described in detail, as shown in Algorithm 1. A two-stage framework is implemented to achieve stable and efficient tracking control. The algorithm first initializes the policy network $\pi_{\phi }$ and collects dataset D using the expert agent (lines 1–6). During the supervised learning phase, state–action pairs are sampled from expert dataset D to minimize the MSE between the policy network’s outputs and the expert’s actions through BC, completing the initial imitation learning of the policy (lines 7–10).
**Algorithm 1** Path tracking based on HSRL.**Input**: trajectory length *L*, pretraining iterations *I*, episode timesteps *T*, learning rate α**Initialize** env, expert agent, offline dataset D, initialized policy $\pi_{\phi }$ with random weights**Phase 1**: Dataset acquisition and offline pretraining  1:Sample the initial state $s_{1}$ from the env  2:**for** each timestep *l* in *L* **do**  3:    $a_{l}\leftarrow \mathrm{expert}\text{agent.get\_action(}s_{l})$;  4:    Execute $a_{l}$, observe reward $r_{l}$ and next state $s_{l+1}$;  5:    Store transition tuple $\left(s_{l},a_{l},r_{l},s_{l+1}\right)$ in offline dataset D;  6:**end for**  7:**for** each iteration *i* in *I* **do**  8:    Sample batch $\left\{\left(s_{i},a_{i}\right)\right\}$ from D  9:    Update ϕ via supervised learning: $\min\sum ∥\pi_{\phi }\left(s_{i}\right)-a_{i}{∥}^{2}$10:**end for****Phase 2**: Online Reinforcement Learning**Initialize** critic networks $Q_{\theta_{1}},Q_{\theta_{2}}$ and target networks $\theta_{1}^{\prime }\leftarrow \theta_{1}$, $\theta_{2}^{\prime }\leftarrow \theta_{2}$, $\phi^{\prime }\leftarrow \phi$ with random weights, replay buffer B11:**for** each timestep *t* in *T* **do**12:    Select action: $a_{t}\leftarrow \pi_{\phi }\left(s_{t}\right)+\epsilon$, ϵ∼N(0,σ)13:    Execute $a_{t}$, observe $r_{t},s_{t+1}$, store $\left(s_{t},a_{t},r_{t},s_{t+1}\right)$ in B14:    Sample mini-batch of *N* transitions $\left(s_{t},a_{t},r_{t},s_{t+1}\right)$ from B15:    Generate target action: ${\tilde{a}}_{t+1}\leftarrow \pi_{\phi^{\prime }}\left(s_{t+1}\right)+\epsilon$, $\epsilon ∼clip(N\left(0,\tilde{\sigma }\right),-c,c)$16:    Compute target value: $y\leftarrow r_{t}+\gamma \min_{i=1,2}Q_{\theta_{i}^{\prime }}\left(s_{t+1},{\tilde{a}}_{t+1}\right)$17:    Update critic: $\theta_{i}\leftarrow \arg\min_{\theta_{i}}\frac{1}{N}\sum {\left(y-Q_{\theta_{i}}\left(s_{t},a_{t}\right)\right)}^{2}$18:    **if** tmodd=0 **then**19:        Compute policy gradient:20:        $\nabla_{\phi }J\leftarrow \frac{1}{N}\sum \nabla_{a_{t}}Q_{\theta_{1}}\left(s_{t},a_{t}\right){|}_{a_{t}∼\pi_{\phi }\left(s_{t}\right)}\nabla_{\phi }\pi_{\phi }\left(s_{t}\right)$21:        Update actor: $\phi \leftarrow \phi +\alpha \nabla_{\phi }J$22:        Soft update target networks:23:        $\theta_{i}^{\prime }\leftarrow \tau \theta_{i}+\left(1-\tau \right)\theta_{i}^{\prime }$24:        $\phi^{\prime }\leftarrow \tau \phi +\left(1-\tau \right)\phi^{\prime }$25:    **end if**26:**end for**

In the online RL phase, the policy network generates action $a_{t}$ based on the current state, and after execution, collects the transition tuple $\left(s_{t},a_{t},r_{t},s_{t+1}\right)$ in the experience replay buffer B (lines 11–13). In the update phase, after sampling the mini-batch of data, the TD target value is calculated, where ${\tilde{a}}_{t+1}$ is generated by the target policy network $\pi_{\phi^{\prime }}$, and clipped noise is added. The parameters of the dual critic networks are updated by minimizing the MSE between the predicted Q-value and the target value (lines 14–17). To suppress the overestimation of Q-values, every *d* steps, the policy network is updated through gradient backpropagation based on the critic network, and soft updates are performed on the target network (lines 18–25). This design combines the stability of supervised learning with the adaptability of online RL, effectively enhancing the robustness and convergence efficiency of the policy through the dual critic mechanism and noise smoothing.

## 4. Experiment

This section comprehensively outlines the experimental setup, selected baselines, assessment protocols, ablation study, hyperparameter sensitivity analysis of the reward function, and empirical results analysis, with the aim of validating the proposed method’s effectiveness and quantifying performance improvements across critical metrics.

### 4.1. Experimental Setup

#### 4.1.1. Implementation Details

All experiments utilized a NVIDIA GeForce RTX 3090 GPU, and the HSRL model was implemented using the PyTorch 2.7.1 framework. All evaluations in this experiment were conducted within the CARLA 0.9.14 environment.

#### 4.1.2. Training Dynamics Analysis

**Figure 5:**
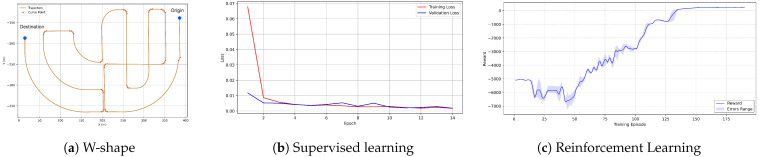
Training setup of the policy in the HSRL process. (**a**) W-shape route used for RL training. (**b**) Supervised learning curve. (**c**) RL curve.

The training process consists of two sequential phases: supervised training via BC and RL. During the supervised training phase, expert demonstrations are collected using an expert agent to construct a dataset from which state–action pairs are extracted for initial policy cloning. The loss reduction curve of the BC procedure is illustrated in Figure 5b. Subsequently, in the RL phase, the agent interacts with the environment within training scenario represented in Figure 5a at speeds ranging from 10 to 120 km/h to acquire rewards for policy updates. The learning curve of this training process is shown in Figure 5c, where the cumulative reward converges after 135 episodes.

#### 4.1.3. Baselines and Evaluation Metrics

Tests were conducted on publicly available predefined paths, where selected comparison methods were maintained under optimal operational conditions throughout testing. Methods include PID based on Wael Farag Tuning Method (WAF-Tune) [26] and MPC-based path tracking [31].

WAF-Tune-based PID represents a novel tuning scheme in the PID framework. It uses an ad hoc trial-and-error-based technique and uses only the CTE as an input to the controller, whereas the output is the steering command. The steering command is produced after applying proportional, integral, and differential control to it in terms of Kp, Ki, and Kd coefficients, respectively. Its main design effort is to carefully tune these three coefficients to obtain the best possible performance. The performance can be simply defined as letting the vehicle follow the predefined path as closely as possible with the lowest aggregated CTE throughout the entire trip. The main goal of the controller is to minimize the aggregated CTE (the objective function), as given by Equation (21). The PID controller was configured with a throttle input of 0.3 and the coefficients (Kp,Ki, and Kd) set to (0.35, 0.0005, and 6.5), which were finalized using WAF-Tune method.

(21) $$\mathrm{Objective}\;\mathrm{Function}=\min_{i=0}^{N}\frac{1}{N}\sum_{i=0}^{N}CTE_{i}^{2}$$

MPC-based path tracking [31] is a classical model-based controller. It employs a well-known bicycle model with four states [x,y,θ,v], as follows:

(22) $$\left\{\begin{array}{cc} \dot{x} & =v\cos(\theta +\beta )\\ \dot{y} & =v\sin(\theta +\beta )\\ \dot{\theta } & =\frac{v}{l_{r}+l_{f}}\sin\left(\beta \right)\\ \dot{v} & =a \end{array}\right.$$

where $\beta =\arctan\left(\frac{l_{r}}{l_{r}+l_{f}}\tan\delta \right)$ denotes the slip angle, and $l_{r}$ and $l_{f}$ are the distances from rear and front axles to the center of vehicles, respectively. θ and *a* are the steering angle of front wheels and acceleration, respectively.

For a specified reference trajectory within the prediction horizon, the objective of the model predictive control system is to minimize the discrepancy between the predicted output and the desired path. This is achieved through an optimized cost function that enables the autonomous vehicle to track the target trajectory with both rapid response and smooth maneuvering. To ensure effective path tracking performance, the controller must simultaneously address system state deviations and optimize control outputs. The resulting objective function for the path-tracking controller is formulated as a quadratic cost function that incorporates both the system states and control inputs:

(23) $$\begin{array}{cc} J\left(k\right)=\sum_{j=1}^{N_{p}} & \left[{\tilde{\chi }}^{T}\left(k+j\right)Q\tilde{\chi }\left(k+j\right)+{\tilde{u}}^{T}\left(k+j-1\right)\tilde{R}\tilde{u}\left(k+j-1\right)\right]\\ s.t.\;\; & u_{\min}\left(t+k\right)\leq u\left(t+k\right)\leq u_{\max}\left(t+k\right),\\ \; & \Delta u_{\min}\left(t+k\right)\leq \Delta u\left(t+k\right)\leq \Delta u_{\max}\left(t+k\right),\\ \; & y_{\min}\left(t+k\right)\leq y\left(t+k\right)\leq y_{\max}\left(t+k\right). \end{array}$$

where *Q* and *R* denote the weighting matrices for control actions and control increments, respectively; the index *k* adopts different ranges depending on the constraint type: $k=0,1,\ldots ,N_{c}-1$ for control and control increment constraints, whereas $k=1,\ldots ,N_{p}$ corresponds to output constraints. The first component in Equation (23) quantifies the system’s tracking performance capability, while the second term enforces regulation on the control sequence. A notable advantage of this objective function formulation is its inherent structure that facilitates direct transformation into a standard quadratic programming (QP) problem.

Moreover, the parameter settings for PID and MPC are consistent with those in the original study. The main parameters of the experiments are referenced in Table 1.

To validate the effectiveness of the model, the experiments evaluated the performance of HSRL in terms of tracking performance and reduction in MS, comparing it with PID and MPC. For path tracking, lateral deviation was selected as the primary evaluation metric, with the reason being that the lateral error directly determines whether the vehicle deviates from the lane or trajectory and serves as the primary factor affecting collision risks.

Jerking and the MSDV are key contributors to MS, where jerking dominates transient discomfort (e.g., sudden braking causing a “lurching sensation”), while the MSDV reflects the cumulative fatigue-inducing stimulation of the vestibular system due to prolonged vibrations (e.g., low-frequency body sway during continuous winding mountain roads).

### 4.2. Tracking Performance

**Figure 6:**
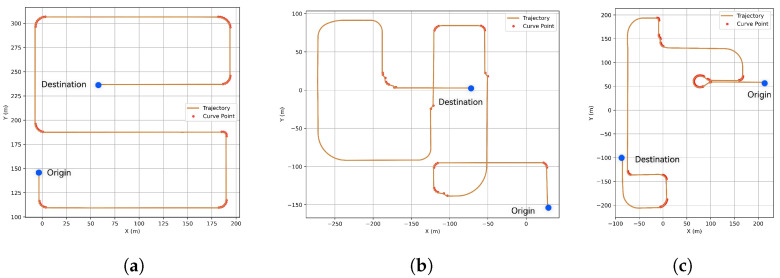
Three test scenarios for tracking and MS performance validation at a speed of 35 km/h and blue dots mark the vehicle’s start and end locations. (**a**) S-shape. (**b**) U-shape. (**c**) O-shape.

The tracking and MS tests were conducted sequentially in S-shape, U-shape, and O-shape routes in Figure 6 at a speed of 35 km/h. The high-speed application is demonstrated later. The S-shape route features single-lane roads and T-shaped intersections, making it suitable for evaluating basic urban driving. In contrast, the O-shape route includes a sharp turn, which tests the vehicle’s steering performance.

Table 2 shows the statistical results of lateral deviation and jerking. It is observed that HSRL achieves consistently high average performance in both training and testing scenarios.

**Figure 7:**
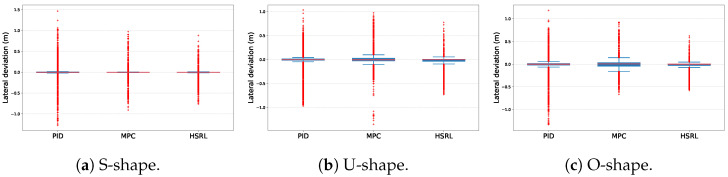
The path tracking comparison results of three scenarios.

The path tracking comparison results are shown in Figure 7. In the simpler test scenario, the S-shape route Figure 6a, the error control performance of all three controllers is better than rest of test scenarios. Additionally, only the HSRL method was able to control the error within a relatively narrow range. With respect to low-error-range control, the PID and HSRL controllers exhibit similar effectiveness in precision tracking. In simple terms, the HSRL model outperformed the PID and MPC controllers in terms of tracking. The excellent tracking performance of the HSRL is due to the data-driven mechanism, which can directly learn the nonlinear dynamic characteristics of complex systems without relying on precise mathematical models, making it highly adaptable.

As presented in Table 3, the maximum lateral deviation error (ME), its occurrence timestep (t), and the corresponding road curvature (k) are detailed. For the PID controller, its fixed gain parameters struggle to adapt to rapidly changing tracking demands in highly dynamic scenarios. The integral term may accumulate significant errors, while the derivative term’s sensitivity to noise can cause oscillatory control outputs, ultimately leading to substantial error values. Although the MPC controller excels in prediction and optimization, its computational delay may become pronounced under complex scenarios. The HSRL controller’s training data may not have fully covered extreme or rare operating conditions, resulting in the maximum lateral deviation error. Nevertheless, it outperformed both PID and MPC.

### 4.3. Reduction in MS Performance

**Figure 8:**
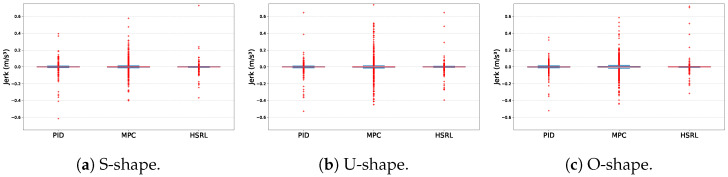
The jerking comparison results of three test scenarios.

**Figure 9:**
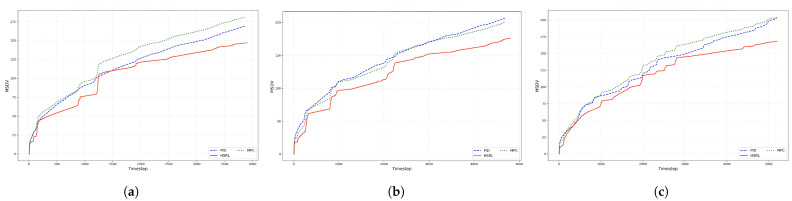
MSDV comparison results in three different routes. (**a**), (**b**) and (**c**) show the cumulative values of MSDV over time for the vehicle in the S-shape, U-shape, and O-shape scenarios, respectively.

To measure the performance of three controllers in reducing passengers’ MS, jerking and the MSDV were considered simultaneously. The experimental results of jerking are shown in Figure 8, and the MSDV comparison results are shown in Figure 9.

With respect to jerks, MPC cannot achieve satisfactory performance in either maximum jerk control or low-jerk-range scenarios. PID and HSRL methods have similar performance, while the HSRL performs better in low-jerk-range scenarios.

In MSDV reduction, the PID and MPC methods have similar performance. Additionally, the HSRL method demonstrates a significant reduction in MSDV values compared to PID and MPC approaches. Based on data from three test scenarios, the HSRL method reduces the MSDV by approximately 15.7%, which is projected to effectively lower the incidence of passenger MS.

Furthermore, it is also observed that as the scenario becomes more complex from the S-shape route to O-shape, the final accumulated MSDV shows a progressive rise. Thanks to the HSRL supporting multi-objective joint optimization, which integrates the standardized MSDV into the reward mechanism, the proposed method performs excellently in reducing passengers’ MSDV.

The synergistic effects of jerking and the MSDV collectively determine passengers’ MS perception. While jerking quantifies transient discomfort caused by abrupt acceleration changes, the MSDV reflects the cumulative vibrational energy exposure over time—a critical factor for prolonged sickness onset. The experimental results demonstrate that HSRL’s ability to simultaneously suppress high-frequency jerks (reducing instantaneous discomfort) and minimize MSDV accumulation (mitigating long-term sickness risk) creates a complementary advantage. Specifically, smoother jerk transitions alleviate acute motion disturbances, whereas the 15.7% MSDV reduction indicates substantially lower cumulative motion energy transmission to passengers. Such comprehensive performance enhancement becomes particularly vital in complex driving scenarios where sharp, intermittent jerks and prolonged irregular vibrations synergistically exacerbate MS severity, highlighting the necessity of multi-objective control frameworks for holistic ride comfort improvement.

### 4.4. High-Speed Performance

**Figure 10:**
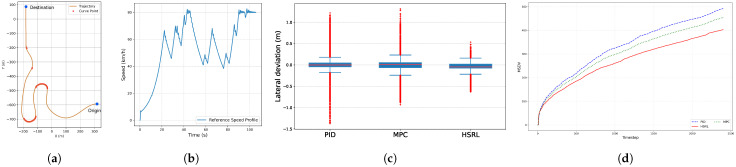
Experimental setup and results for high-speed tests. (**a**) Random shape. (**b**) Speed profile. (**c**) Lateral deviation results. (**d**) MSDV results.

The high-speed performance evaluation was conducted under a unified reference speed profile, as shown in Figure 10b, which required rapid acceleration to peak speeds followed by deceleration before sharp turns. The test scenario (Figure 10a) included multiple curve points to test path tracking robustness. The PID, MPC, and the proposed HSRL were compared based on lateral deviation (Figure 10c) and the MSDV over time (Figure 10d).

Regarding path tracking, PID exhibited the largest lateral deviations with pronounced fluctuations, while MPC showed moderate improvements but still struggled with sharp curves. In contrast, HSRL demonstrated the smallest overall deviations, with a tighter distribution and fewer extreme values. For the MSDV, PID and MPC showed gradual increases, but HSRL maintained the lowest cumulative values throughout the experiment. Notably, HSRL achieved superior path tracking stability while minimizing passenger discomfort, outperforming both PID and MPC in balancing high-speed dynamics and ride smoothness. The superior performance of HSRL in high-speed scenarios is attributed to its multi-objective optimization framework and inherent robustness, which enable precise path tracking while minimizing passenger discomfort.

### 4.5. Reward Hyperparameter Sensitivity Analysis

**Figure 11:**
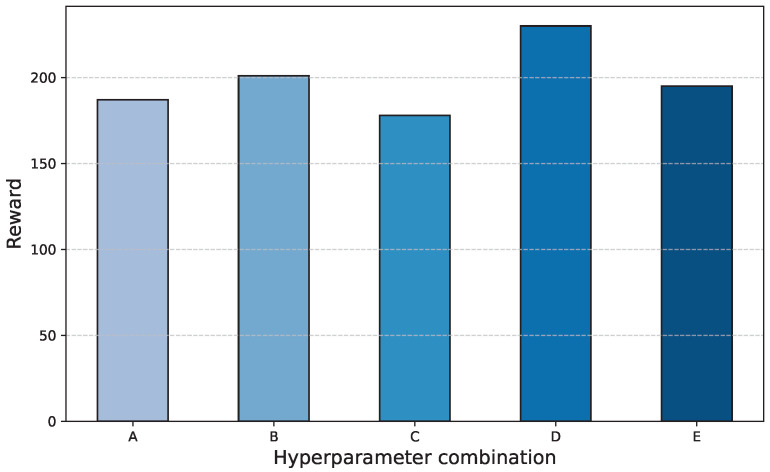
Reward hyperparameter sensitivity analysis.

To investigate the impact of the reward weight hyperparameters on algorithm performance, a sensitivity analysis was conducted, as shown in Figure 11. The x-axis represents different parameter sets, with specific hyperparameter values detailed in Table 4. The y-axis indicates the corresponding reward scores for these parameter sets, representing the overall performance of the method. All subsequent experiments in this paper were implemented using the highest-scoring configuration, D.

Compared to D, configuration A increases the speed reward coefficient, forcing the vehicle to maintain high speed in curves and seriously compromising path tracking accuracy, ultimately leading to a decrease in total reward. Configuration B enhances the weight of control components, resulting in a decline in the reward. This is because overemphasizing steering smoothness causes delayed steering responses during sharp turns, increasing tracking errors. Configurations C and E, respectively, amplify the weights of tracking error and the MSDV, both resulting in reduced rewards. This occurs because overemphasizing either tracking error or the MSDV negatively impacts the other metric.

### 4.6. Ablation Study

To comprehensively evaluate HSRL’s performance across different components, this study designed a series of ablation experiments. In these experiments, specific components of HSRL were removed sequentially, and the resulting models were evaluated in three test scenarios shown in Figure 6.

To evaluate the overall performance of the resulting models, the test data from three scenarios were aggregated by calculating the mean, standard deviation, maximum value, and Total Motion Sickness Dose Value (TMSDV). The mean quantifies the central tendency of the system’s behavior, the standard deviation reflects the consistency across scenarios, the maximum value highlights potential performance limits, and the TMSDV serves as a quantitative metric for assessing the impact of vehicle dynamic control on passenger comfort. These aggregated results are summarized in Table 5.

In the experimental data, it can be observed that when the supervised learning objective is removed, the model HSRL w/o $L^{SL}$ exhibits significant performance degradation across all metrics compared to the full HSRL model. Specifically, the following were found:

- Lateral deviation increases from 0.0925 m (mean) to 0.1468 m, with a 41.3% increase in standard deviation.
- Jerk rises from 0.0202 m/$s^{3}$ (mean) to 0.0315 m/$s^{3}$, accompanied by a 20.8% increase in standard deviation.
- TMSDV increases from 475.7 to 681.3, with a 43.2% increase.

In contrast, the HSRL w/o $L^{RL}$ shows moderate degradation (e.g., TMSDV increases by 20.4%), indicating that RL contributes to adaptability but is less essential than supervised learning for core accuracy and comfort. This further confirms that supervised learning component is critical for foundational performance, while RL provides complementary adaptability.

## 5. Conclusions

This paper proposes an innovative HSRL framework, which employs a two-stage optimization mechanism. In the first stage, an initial policy is trained via BC to rapidly acquire fundamental knowledge. In the second stage, the model is refined through RL, enabling autonomous exploration to achieve superior performance. For RL training, HSRL leverages the TD3 algorithm, ensuring both operational efficiency and robust safety guarantees for the AD system. Validated through experiments across multiple test scenarios, the HSRL method demonstrates outstanding path-tracking performance and generalization capability while reducing the MSDV by 15.7% during AD operations.

Looking ahead to future research directions, the proposed HSRL framework still holds significant development potential. In MS dynamics, while this study quantifies MS via acceleration metrics, future investigations will integrate vehicle-specific dynamics, including suspension characteristics and body resonance frequencies, to establish a multi-physics discomfort model. Additionally, field tests under heterogeneous road conditions and sensor noise profiles are essential. A phased implementation framework will be designed to address latency tolerance and edge computing constraints in physical vehicle platforms.

## Figures and Tables

**Table 1:** Main parameters used in the experiments.

Parameters	Value
Supervised Learning	
Learning rate	1 × $10^{-3}$
Policy noise	0.1
Batch size	64
Optimizer	Adam
Reinforcement Learning	
Optimizer	Adam
Policy frequency	2
Policy noise	0.2
Policy learning rate	3 × $10^{-4}$
Discount factor γ	0.99
tau	0.005
Reward function weights $\alpha_{1},\alpha_{2},\alpha_{3},\beta_{1},\beta_{2}$	1.5, 1.0, 2.5, 0.1, 1.6
PID based on WAF-Tune	
35 km/h (Constant speed)–Lateral (Kp, Ki, Kd)	(0.35, 0.0005, 6.5)
High speed–Lateral (Kp, Ki, Kd)	(0.2, 0.0000, 7.0)
High speed–Longitudinal (Kp, Ki, Kd)	(0.3, 0.0500, 0.5)
MPC	
Sample time (s)	0.05
Prediction horizon	20
Control horizon	5
Vehicle mass (kg)	1720
Front suspension stiffness (N/m)	35,000
Rear suspension stiffness (N/m)	30,000

**Table 2:** Lateral deviation and jerking on different routes.

Scenario	Lateral Deviation (Mean, Std, Max)	Jerk (Mean, Std, Max)
W-shape (training)	(0.0549, 0.0815, 0.4443)	(0.0194, 0.0551, 0.5834)
S-shape (test)	(0.0469, 0.0809, 0.7908)	(0.0189, 0.0571, 0.6745)
U-shape (test)	(0.1219, 0.1587, 0.8659)	(0.0210, 0.0559, 0.6009)
O-shape (test)	(0.1159, 0.1354, 0.7523)	(0.0204, 0.0602, 0.6714)

**Table 3:** The point of maximum error.

Method	S- Shape (ME, t, k)	U- Shape (ME, t, k)	O- Shape (ME, t, k)
PID	(1.47, 2743, 0.018)	(1.07, 2973, 0.014)	(1.23, 429, 0.027)
MPC	(0.98, 3208, 0.014)	(0.97, 2970, 0.013)	(0.84, 438, 0.025)
HSRL	(0.79, 3215, 0.015)	(0.87, 1988, 0.017)	(0.75, 429, 0.026)

**Table 4:** Hyperparameter combination.

Hyperparameter Combination	Value
A	$\alpha_{1}=2.0,\alpha_{2}=1.0,\alpha_{3}=2.5,\beta_{1}=0.1,\beta_{2}=1.6$
B	$\alpha_{1}=1.5,\alpha_{2}=1.0,\alpha_{3}=2.5,\beta_{1}=0.2,\beta_{2}=1.6$
C	$\alpha_{1}=1.5,\alpha_{2}=1.5,\alpha_{3}=3.0,\beta_{1}=0.1,\beta_{2}=1.6$
D	$\alpha_{1}=1.5,\alpha_{2}=1.0,\alpha_{3}=2.5,\beta_{1}=0.1,\beta_{2}=1.6$
E	$\alpha_{1}=1.5,\alpha_{2}=1.0,\alpha_{3}=2.5,\beta_{1}=0.1,\beta_{2}=2.0$

**Table 5:** Ablation study results on the objective terms.

Method	Lateral Deviation (Mean, Std, Max)	Jerk (Mean, Std, Max)	TMSDV
HSRL	(0.0925, 0.1185, 0.8659)	(0.0202, 0.0578, 0.6745)	475.7
HSRL w/o $L^{SL}$	(0.1468, 0.1676, 0.9817)	(0.0315, 0.0687, 0.9719)	681.3
HSRL w/o $L^{RL}$	(0.1381, 0.1538, 0.8732)	(0.0293, 0.0613, 0.89324)	572.8

## Data Availability

The data presented in this study are available upon request from the corresponding author.

## References

[1] Parekh D., Poddar N., Rajpurkar A., Chahal M., Kumar N., Joshi G.P., Cho W. (2022). A review on autonomous vehicles: Progress, methods and challenges. Electronics.

[2] Pettersson I., Karlsson I.M. (2015). Setting the stage for autonomous cars: A pilot study of future autonomous driving experiences. IET Intell. Transp. Syst..

[3] Li Q., Chen L., Li M., Shaw S.L., Nüchter A. (2013). A sensor-fusion drivable-region and lane-detection system for autonomous vehicle navigation in challenging road scenarios. IEEE Trans. Veh. Technol..

[4] Chen L., Fan L., Xie G., Huang K., Nüchter A. (2017). Moving-object detection from consecutive stereo pairs using slanted plane smoothing. IEEE Trans. Intell. Transp. Syst..

[5] Fu Y., Li C., Yu F.R., Luan T.H., Zhang Y. (2020). A decision-making strategy for vehicle autonomous braking in emergency via deep reinforcement learning. IEEE Trans. Veh. Technol..

[6] Chen L., Shan Y., Tian W., Li B., Cao D. (2018). A fast and efficient double-tree RRT*-like sampling-based planner applying on mobile robotic systems. IEEE/ASME Trans. Mechatron..

[7] Yao Q., Tian Y., Wang Q., Wang S. (2020). Control strategies on path tracking for autonomous vehicle: State of the art and future challenges. IEEE Access.

[8] Bertolini G., Straumann D. (2016). Moving in a moving world: A review on vestibular motion sickness. Front. Neurol..

[9] Reason J.T. (1978). Motion sickness adaptation: A neural mismatch model. J. R. Soc. Med..

[10] Medina Santiago A., Orozco Torres J.A., Hernández Gracidas C.A., Garduza S.H., Franco J.D. (2023). Diagnosis and Study of Mechanical Vibrations in Cargo Vehicles Using ISO 2631-1: 1997. Sensors.

[11] Wada T. (2016). Motion sickness in automated vehicles. Advanced Vehicle Control.

[12] Diels C., Bos J.E. (2016). Self-driving carsickness. Appl. Ergon..

[13] Paden B., Čáp M., Yong S.Z., Yershov D., Frazzoli E. (2016). A survey of motion planning and control techniques for self-driving urban vehicles. IEEE Trans. Intell. Veh..

[14] Amer N.H., Zamzuri H., Hudha K., Aparow V.R., Kadir Z.A., Abidin A.F.Z. (2016). Modelling and trajectory following of an armoured vehicle. Proceedings of the 2016 SICE International Symposium on Control Systems (ISCS).

[15] Zhao P., Chen J., Song Y., Tao X., Xu T., Mei T. (2012). Design of a control system for an autonomous vehicle based on adaptive-pid. Int. J. Adv. Robot. Syst..

[16] Park M.W., Lee S.W., Han W.Y. (2014). Development of lateral control system for autonomous vehicle based on adaptive pure pursuit algorithm. Proceedings of the 2014 14th International Conference on Control, Automation and Systems (ICCAS 2014), Gyeonggi-do, Republic of Korea.

[17] Hoffmann G.M., Tomlin C.J., Montemerlo M., Thrun S. (2007). Autonomous automobile trajectory tracking for off-road driving: Controller design, experimental validation and racing. Proceedings of the 2007 American Control Conference.

[18] Sharp R. (2012). Rider control of a motorcycle near to its cornering limits. Veh. Syst. Dyn..

[19] Yamashita A.S., Alexandre P.M., Zanin A.C., Odloak D. (2016). Reference trajectory tuning of model predictive control. Control Eng. Pract..

[20] Falcone P., Borrelli F., Asgari J., Tseng H.E., Hrovat D. (2007). Predictive active steering control for autonomous vehicle systems. IEEE Trans. Control Syst. Technol..

[21] Gutjahr B., Gröll L., Werling M. (2016). Lateral vehicle trajectory optimization using constrained linear time-varying MPC. IEEE Trans. Intell. Transp. Syst..

[22] Siddiqi M.R., Milani S., Jazar R.N., Marzbani H. (2022). Motion sickness mitigating algorithms and control strategy for autonomous vehicles. IEEE Trans. Intell. Transp. Syst..

[23] Amer N.H., Zamzuri H., Hudha K., Kadir Z.A. (2017). Modelling and control strategies in path tracking control for autonomous ground vehicles: A review of state of the art and challenges. J. Intell. Robot. Syst..

[24] Shan Y., Yang W., Chen C., Zhou J., Zheng L., Li B. (2015). CF-pursuit: A pursuit method with a clothoid fitting and a fuzzy controller for autonomous vehicles. Int. J. Adv. Robot. Syst..

[25] Zhu Q., Huang Z., Liu D., Dai B. (2016). An adaptive path tracking method for autonomous land vehicle based on neural dynamic programming. Proceedings of the 2016 IEEE International Conference on Mechatronics and Automation.

[26] Farag W. (2020). Complex trajectory tracking using PID control for autonomous driving. Int. J. Intell. Transp. Syst. Res..

[27] Park M., Lee S., Han W. (2015). Development of steering control system for autonomous vehicle using geometry-based path tracking algorithm. Etri J..

[28] Lee D., Lee S.J., Yim S.C. (2020). Reinforcement learning-based adaptive PID controller for DPS. Ocean Eng..

[29] Ghafarian M., Watson M., Mohajer N., Nahavandi D., Kebria P.M., Mohamed S. (2023). A review of dynamic vehicular motion simulators: Systems and algorithms. IEEE Access.

[30] Zha Y., Deng J., Qiu Y., Zhang K., Wang Y. (2023). A survey of intelligent driving vehicle trajectory tracking based on vehicle dynamics. SAE Int. J. Veh. Dyn. Stab. NVH.

[31] Chen S., Chen H., Negrut D. (2020). Implementation of MPC-based path tracking for autonomous vehicles considering three vehicle dynamics models with different fidelities. Automot. Innov..

[32] Mattingley J., Boyd S. (2012). CVXGEN: A code generator for embedded convex optimization. Optim. Eng..

[33] Merabti H., Belarbi K., Bouchemal B. (2016). Nonlinear predictive control of a mobile robot: A solution using metaheuristcs. J. Chin. Inst. Eng..

[34] Shladover S.E., Desoer C.A., Hedrick J.K., Tomizuka M., Walrand J., Zhang W.B., McMahon D.H., Peng H., Sheikholeslam S., McKeown N. (1991). Automated vehicle control developments in the PATH program. IEEE Trans. Veh. Technol..

[35] Kapania N.R., Gerdes J.C. (2015). Design of a feedback-feedforward steering controller for accurate path tracking and stability at the limits of handling. Veh. Syst. Dyn..

[36] Ly A.O., Akhloufi M. (2020). Learning to drive by imitation: An overview of deep behavior cloning methods. IEEE Trans. Intell. Veh..

[37] Chitta K., Prakash A., Jaeger B., Yu Z., Renz K., Geiger A. (2022). Transfuser: Imitation with transformer-based sensor fusion for autonomous driving. IEEE Trans. Pattern Anal. Mach. Intell..

[38] Ye Y., Qiu D., Wang H., Tang Y., Strbac G. (2021). Real-time autonomous residential demand response management based on twin delayed deep deterministic policy gradient learning. Energies.

[39] Puterman M.L. (1990). Markov decision processes. Handbooks Oper. Res. Manag. Sci..

[40] Golding J., Markey H., Stott J. (1995). The effects of motion direction, body axis, and posture on motion sickness induced by low frequency linear oscillation. Aviat. Space Environ. Med..

[41] Donohew B.E., Griffin M.J. (2004). Motion sickness: Effect of the frequency of lateral oscillation. Aviat. Space, Environ. Med..

